# Soziale Isolation im Homeoffice im Kontext der COVID-19-Pandemie: Hinweise für die Gestaltung von Homeoffice im Hinblick auf soziale Bedürfnisse

**DOI:** 10.1007/s40664-020-00410-w

**Published:** 2020-10-23

**Authors:** Julia Christine Lengen, Ann-Christin Kordsmeyer, Elisabeth Rohwer, Volker Harth, Stefanie Mache

**Affiliations:** Zentralinstitut für Arbeitsmedizin und Maritime Medizin (ZfAM), Universitätsklinikum Hamburg Eppendorf (UKE), Seewartenstraße 10, Haus 1, 20459 Hamburg, Deutschland

**Keywords:** Corona-Pandemie, Digitale Zusammenarbeit, Telearbeit, Virtuelle Teams, Gesundheitsförderung, Corona pandemic, Telecommuting, Telework, Virtual teams, Health promotion

## Abstract

**Hintergrund:**

In der von Kontaktbeschränkungen geprägten Hochphase der COVID-19-Pandemie ermöglichen viele Unternehmen ihren Beschäftigten aus Gründen des Infektionsschutzes das Arbeiten aus dem Homeoffice.

**Fragestellung:**

In dieser Literaturübersicht wird der Frage nachgegangen, wie Arbeit im Homeoffice und somit die digitale Zusammenarbeit in Teams möglichst gesundheitsfördernd und sozialer Isolation vorbeugend gestaltet werden kann.

**Ergebnisse:**

Die bestehende arbeitspsychologische Forschung benennt als Grundvoraussetzung für Zusammenarbeit von ortsunabhängigen Teams angemessene, bestenfalls angereicherte, Informations- und Kommunikationsmedien, begleitet von ausreichend und verständlicher technischer Unterstützung. Auch eine stetige, sozial unterstützende Kommunikation innerhalb der Teams und mit der Führungskraft sowie gesundheitsfördernde Führung haben einen positiven Einfluss auf die psychische Gesundheit der Beschäftigten. Ergänzend werden individuelle (digitale) Gesundheitsförderungsmaßnahmen und flexible Arbeitszeiten empfohlen.

**Fazit:**

Diese aus der Literatur abgeleiteten multifaktoriellen Maßnahmenansätze werden Betrieben mit Beschäftigten im Homeoffice vorgeschlagen, um die arbeitsbezogenen, gesundheitsgefährdenden Auswirkungen der Krise – vor allem hinsichtlich sozialer Isolation – abzumildern und die Gesundheit ihrer Beschäftigten zu fördern.

## Abstract (en)

**Background:**

During the peak times of the COVID-19 pandemic, which were characterized by contact restrictions, many companies initiated telework for their employees due to infection prevention.

**Objective:**

In this literature review working from home and therefore digital cooperation in a virtual team was investigated, focusing on the organization of occupational health promotion aspects in the context of prevention of social isolation.

**Results:**

The current occupational health psychology research identified appropriate and enriched information and communication media accompanied by sufficient and understandable technical support as basic prerequisites for the collaboration of location-independent teams. Also, a continuous socially supporting communication within the team and with the supervisor as well as health-promoting leadership have a positive impact on the employees’ mental health. Additionally, individual (digital) health promotion interventions and flexible working hours are recommended.

**Conclusion:**

These multifactorial approaches to measures derived from the literature are suggested for companies with employees working predominantly from home to reduce work-related adverse health effects from the crisis, especially with respect to social isolation and to promote their employees’ health.

Seit Beginn der COVID-19-Pandemie im März 2020 in Deutschland ermöglichen viele Betriebe ihren Beschäftigten das Arbeiten im Homeoffice [13]. Neben den gesellschaftlichen Kontaktbeschränkungen vermindert das überwiegende Arbeiten von zu Hause auch die sozialen Kontakte im Kollegenkreis. Mit zunehmender Dauer und Intensität der Heimarbeit können Beschäftigte somit von nachteiligen Faktoren wie dem steigenden Risiko der sozialen Isolation betroffen sein [2]. Derzeit liegen diesbezüglich keine belastbaren arbeitspsychologischen Erkenntnisse aus der Zeit der COVID-19-Pandemie vor. Daher können für die Gestaltung einer möglichst gesundheitsfördernden Arbeitsorganisation, die sozialer Isolation dieser Zielgruppe entgegenwirkt, lediglich Rückschlüsse aus den bisherigen Forschungserkenntnissen der virtuellen Team- und Telearbeit gezogen werden. Diese bilden die aktuelle komplexe Situation jedoch nicht in Gänze ab.

## Homeoffice in Folge der pandemiebedingten Kontaktbeschränkungen in Deutschland

Zur Kontrolle der COVID-19-Pandemie ist die in Deutschland lebende Bevölkerung zeitweise dazu angehalten, alle nicht zwingend notwendigen Kontakte zu vermeiden. In dem Beschluss „*Beschränkungen des öffentlichen Lebens zur Eindämmung der COVID-19-Epidemie*“ von der Bundeskanzlerin und Regierungschef/innen der Länder vom 15. April 2020 werden Unternehmen dazu aufgefordert, im Arbeitskontext die Möglichkeiten der Beschäftigung aus dem Homeoffice auszuschöpfen. Daraufhin ermöglichten viele Betriebe ihren Beschäftigten das Arbeiten von Zuhause [13]. Umfragen zufolge arbeiteten in Hochzeiten der COVID-19-Pandemie im März und April 2020 bis zu 26,5 % der Beschäftigten – gegenüber 12 % in der Zeit vor der Krise – im Homeoffice [38]. Diese Präventionsmaßnahme dient neben dem Schutz der Bevölkerung auch der individuellen Infektionsvorbeugung der Beschäftigten und reduziert damit das Ausfallrisiko für Betriebe [10].

Arbeiten im Homeoffice birgt neben einigen gesundheitsförderlichen Aspekten (wie z. B. flexibel gestaltbare Arbeitszeiten oder erhöhte Handlungsspielräume, welche zu höherer Arbeitszufriedenheit, Motivation und Leistungsfähigkeit führen können [16, 17, 19, 29, 39, 44]) jedoch auch nachteilige Faktoren, wie das steigende Risiko der sozialen Isolation [2]. Bentley et al. [7] stellten isolationsbedingt einen signifikanten Anstieg des Stresserlebens sowie einen Abfall der Arbeitszufriedenheit mit zunehmender Arbeitszeit der Beschäftigten im Homeoffice fest [7]. Laut einem Review von Tavares [44] kann eine längere Arbeitstätigkeit ohne soziale Interaktionen zu Einsamkeit und Isolation sowie zu Depressionen führen [44]. Soziale Isolation allgemein kann sowohl für ältere als auch für jüngere Menschen mit verschiedenen negativen Konsequenzen für die psychische Gesundheit wie Depressionen und Angststörungen verbunden sein [4, 6, 42].

Arbeiten im Homeoffice kann im COVID-19-Kontext zu Risiken der sozialen Isolation führen

In dieser Literaturübersicht wird der Frage nachgegangen, wie Arbeit im Homeoffice in Zeiten von pandemiebedingten Kontaktbeschränkungen möglichst gesundheitsfördernd und sozialer Isolation vorbeugend gestaltet werden kann. Mittels einer explorativen Literaturrecherche werden bisher verfügbare arbeitspsychologische Erkenntnisse der Gestaltung von Homeoffice-Arbeitsplätzen aus den Bereichen der virtuellen Team- und Telearbeit recherchiert und zusammengefasst. Auf Basis dieser Forschungserkenntnisse werden Handlungsempfehlungen für Unternehmen zur interaktionsanregenden und gesundheitsfördernden Gestaltung von Homeoffice-Arbeitsplätzen während der COVID-19-Pandemie gegeben.

## Forschungserkenntnisse und abgeleitete Handlungsempfehlungen

Bentley et al. [7] legen nahe, dass soziale Isolation im Rahmen der Beschäftigung im Homeoffice begünstigt wird, wenn keine ausreichende Unterstützung vorliegt [7]. Diese Unterstützung umfasst neben technischem Support und Vertrauen der Unternehmensleitung und Führungskräfte auch die Koordinierung von Aktivitäten sowie die Zusammenarbeit mit Kolleg/innen.

Mangelnde soziale Unterstützung begünstigt soziale Isolation im Homeoffice

Auf Basis des aktuellen Forschungsstandes sind Hinweise auf betriebliche Unterstützungsmaßnahmen, die der sozialen Isolation von Beschäftigten im Homeoffice entgegenwirken können, ableitbar.

### Technische Unterstützung

Grundsätzlich bieten bereitgestellte technische Geräte und Medien den im Homeoffice Beschäftigten die Möglichkeit zur Kontaktpflege mit Kolleg/innen in arbeitsbezogenen Netzwerken. Sie stellen die Grundvoraussetzung für flexible (orts- und teilweise zeitunabhängige) Kommunikation dar, womit Beschäftigte der sozialen Isolation bedarfsgerecht entgegenwirken können [33]. Aus der Forschung können einige Hinweise für die Ausgestaltung und den Umgang mit technischer Ausrüstung sowie Informations- und Kommunikationstechnologien für Homeoffice-Arbeitsstrukturen abgeleitet werden. Es sollten allen Beschäftigten angemessene und funktionierende technische Informations- und Kommunikationsmedien bereitgestellt werden, die flexible und unmittelbare Kommunikation (z. B. mit Kolleg/innen) ermöglichen [33] und möglichst leicht anwendbar sind. Zudem ist die rechtzeitige Bereitstellung von ausreichend verständlichen Informationen zur Funktionsweise von Technologie und IT-System [7] sowie das Gewähren von technischer Unterstützung (bspw. durch einen Serviceanbieter) notwendig [7].

### Soziale Unterstützung und Kommunikation im digital zusammenarbeitenden Team

Mehrere Studien weisen darauf hin, dass soziale Interaktionen im Arbeitskontext einen positiven Effekt sowohl auf das Stressempfinden als auch das Wohlbefinden ausüben [11, 12, 14]. Darüber hinaus kann mit zunehmendem Arbeitszeitanteil im Homeoffice die Beziehungsqualität zwischen Kolleg/innen und somit auch die Arbeitsatmosphäre negativ beeinflusst werden [16]. Die derzeit verfügbare Informations- und Kommunikationstechnologie ermöglicht – bei Ausstattung aller Beschäftigten mit funktionierenden Systemen [33] – die Pflege arbeitsbezogener und sozialer Kontakte über die Entfernung. Um eine gesundheitsförderliche, stetige Kommunikation im digital zusammenarbeitenden Team zu erreichen, bietet es sich an, eine regelmäßige mediengestützte Kommunikation in die Arbeitsstrukturen einzubetten, z. B. durch virtuelle Teamsitzungen [7, 28, 33]. Auch das Fördern der bestehenden kollegialen Beziehungen und Ermutigen zur Interaktion zwischen den Teammitgliedern [20] erweist sich als wertvoll, um einen stetigen Austausch zwischen den im Homeoffice Beschäftigten aufrechtzuerhalten [44]. Zudem können so der Wissensaustausch beibehalten [2], die Arbeitsbeziehungen gepflegt [33], das Teamgefühl erhalten [7, 33], das Netzwerk und das gegenseitige Vertrauen im Team sowie zur Führungskraft aufrechterhalten werden [29, 31].

Vorteilhaft ist die Nutzung synchroner, angereicherter Kommunikationswege (wie z. B. Videotelefonie, bei der nonverbale Interaktionen sichtbar werden oder Telefon mit verbalem Austausch anstatt nur schriftlichem Austausch via E‑Mail; [15]). So können ein empathisches Aufeinandereingehen, Ruhe und emotionale Verbundenheit vermittelt werden, an denen es durch reduzierte Kontakte gerade in der Pandemiezeit eventuell mangelt [26]. Zudem kann dadurch die wahrgenommene mentale Distanz zwischen den im Homeoffice Tätigen reduziert werden [15, 43, 46, 47]. Um kollegiale Unterstützung zu fördern [7], können Ziele und Aufgaben von der Führungskraft interdependent angelegt werden. So wird Zusammenarbeit – auch im Sinne des sozialen Austausches – gefördert und gegenseitige Unterstützung angeregt [24]. Für kollektive Erfolge empfehlen Hertel und Lauer [21] die Nutzung von Anreizen oder belohnenden Aktivitäten [21]. Eine wertschätzende, empathische und offene Kommunikation der Führungskräfte hilft, Missverständnissen vorzubeugen und ein gutes Arbeitsklima trotz räumlicher Distanz zu ermöglichen [24]. Den Beschäftigten sollte zudem Interesse an ihrem Wohlbefinden signalisiert werden [7].

Die Nutzung synchroner, angereicherter Kommunikationsmedien ist von Vorteil

Um einer wahrgenommenen sozialen Isolation entgegenzuwirken und das psychische Beanspruchungserleben der Beschäftigten positiv zu beeinflussen, können auch soziale und organisatorische Unterstützungsangebote seitens des Unternehmens einbezogen werden [7]. So empfiehlt sich etwa eine betriebliche Hilfestellung bei Problemen [7], je nach Betriebsgröße und -struktur, bspw. durch Ansprechpartner/innen für Herausforderungen bzgl. virtueller Teamarbeit, betriebliche Sozialberatung, Employee-Assistance Programs, Rundschreiben, psychologische und/oder soziale Beratung zu arbeitsbezogenen Themen (z. B. soziale Isolation, Kurzarbeit, Jobunsicherheit, Arbeits-Familien-Konflikt etc.).

### Gesundheitsförderliche Führung von digital zusammenarbeitenden Teams

Ein wichtiger Unterstützungsfaktor für Beschäftigte ist die soziale Unterstützung durch die Führungskraft.

Ein enger Kontakt zur Führung, Informationsaustausch, Feedback und Vertrauen sind entscheidend

Dabei spielen ein enger Kontakt zur Führungskraft, Informationsaustausch sowie Feedback und Vertrauen eine entscheidende Rolle [7]. Letzteres stellt die Basis für eine erfolgreiche virtuelle Zusammenarbeit dar und wirkt sich in ortsunabhängig arbeitenden Teams stärker auf die Leistungsfähigkeit aus, verglichen mit traditioneller Zusammenarbeit vor Ort [8]. Um das Vertrauen zu fördern, sollten zum einen aufgabenbezogene Elemente, wie eine transparente Weitergabe von Informationen oder die Berücksichtigung von Vereinbarungen und Verantwortungsbereichen berücksichtigt werden. Diese sollten durch teambezogenen Faktoren (z. B. durch gegenseitiges Feedback oder dem Austausch von privaten Hintergrundinformationen) ergänzt werden [9]. Zum informellen oder formellen Austausch können Chaträume oder mediengestützte Besprechungen dienen. Vor dem Hintergrund einer möglichen sozialen Isolation sollten Führungskräfte eingeräumte Zeiträume für einen informellen, privaten Austausch oder digitale Teambuilding-Maßnahmen [29], wie z. B eine virtuelle Kaffeepause, eindeutig befürworten und ggf. anregen [9]. Zusätzlich berichten Lindner und Greff [35], dass größere Arbeitspakete ein höheres Maß an Vertrauen erfordern [35]. Führungskräfte sollten sich außerdem ihrer gesundheitsbezogenen Vorbildfunktion (bspw. hinsichtlich der Einhaltung von Vereinbarung, Arbeitszeiten oder Erreichbarkeit) bewusst sein, welche sich auf das Verhalten und die Arbeitszufriedenheit der Beschäftigten auswirken kann [41].

Darüber hinaus zeigen Forschungsergebnisse, dass Führungskräfte in der digitalen Zusammenarbeit vermehrt partizipative bzw. unterstützende Rahmenbedingungen implementieren sollten, in denen Teams ihren Austausch und die Arbeitsprozesse mitgestalten können [3, 21, 22]. Die Definition von Aufgaben und deren Verteilung sowie die Festlegung von Zielen und Strategien sollten koordiniert und über passende Informations- und Kommunikationsformate vermittelt werden [1, 3, 21]. So eignen sich bspw. E‑Mails für die Weitergabe von Informationen, während für Ideensammlungen oder Diskussionen angereicherte Medien herangezogen werden sollten [34]. Tiefergehende Informationen zur Zusammenarbeit und Führung virtueller Teams können den Publikationen von Kordsmeyer et al. [30, 31] entnommen werden.

### Individuell gesundheitsförderndes Verhalten der Beschäftigten im Homeoffice

Ort- und zeitflexible Arbeitsformen wie die Arbeit im Homeoffice erfordern von den Beschäftigten ausgeprägte Fähigkeiten zur Selbstorganisation und Disziplin [32]. Es erscheint daher sinnvoll, den Schulungsbedarf hinsichtlich arbeitsorganisatorischer Kompetenzen zu ermitteln, anzupassen und fortlaufend zu evaluieren.

**Figure 1:**
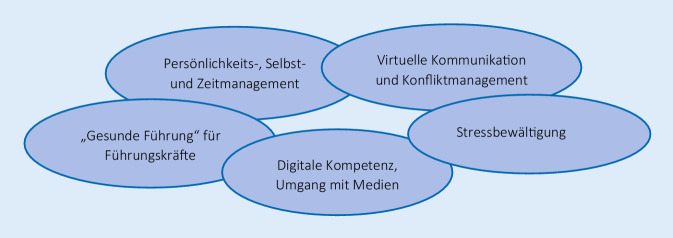

Unternehmen können beim Aufbau individueller und sozialer Ressourcen eine treibende Kraft sein und so negative Beanspruchungsfolgen verhindern bzw. reduzieren und die psychische Gesundheit der im Homeoffice Beschäftigten fördern. Online-Schulungen zum gesundheitsförderlichen Umgang mit der Tätigkeit im Homeoffice könnten u. a. zu den in Abb. 1 dargestellten Themen unterstützend sein.

Unternehmen können durch den Ausbau von Ressourcen die Gesundheit der Beschäftigten fördern

Neben den Aspekten der Arbeitsgestaltung im Homeoffice können Gesundheit und Resilienz der Beschäftigten durch einen entsprechenden Lebensstil gefördert werden. Der Erhalt von Bewegung und Sport hat ein hohes Potenzial, Stressreaktionen zu verringern [37] und birgt Präventionspotenziale bspw. vor Angststörungen und Depressionen [6, 23]. Aufgrund eingeschränkter Bewegungsmöglichkeiten in der Freizeit oder z. B. auf dem Arbeitsweg gewinnt eine gesunde Ernährung noch mehr an Relevanz, um der Gewichtzunahme vorzubeugen. Darauf deuten erste Ergebnisse einer nicht repräsentativen Erhebung hin, laut der über 50 % der französischen Bevölkerung während der COVID-19-bedingten Ausgangssperre im Frühjahr 2020 [25] und rund 20 % der Deutschen bis Ende April 2020 an Körpergewicht zugenommen haben [5]. Auch Maßnahmen zur Schlafhygiene wie eine Schlaf-Wach-Rhythmusstrukturierung oder kognitive Techniken zur Reduktion nächtlicher Grübeleien dienen als Präventionsstrategie für Depressionen [40]. Sofern Aktivitäten schwerfallen, sollte auf eine Tagesstrukturierung geachtet werden. Diese stellt einen wichtigen Teil von Therapien bspw. während depressiver Episoden dar [36]. Mit der Pflege sozialer Beziehungen [26] über digitale Medien kann Einsamkeit und sozialer Isolation begegnet werden.

Führungskräfte können an der Erweiterung der Gesundheitskompetenzen ihrer Beschäftigten mitwirken, sie für diese Gesundheitsthemen sensibilisieren [31] und gesunde Verhaltensweisen in Pausen bzw. der Freizeit anregen. Durch das Bereitstellen gesundheitsförderlicher Rahmenbedingungen, die den Handlungsspielraum erweitern, wie bspw. Gleitzeit oder flexible Pausengestaltung [39], aber auch die Pflege einer vertrauensvollen Arbeitsatmosphäre [20, 31] können Betriebe ihre Beschäftigten bei der Bewältigung der Krisensituation unterstützen. Zudem können gerade Angebote der digitalen Gesundheitsförderung Beschäftigte im Homeoffice erreichen. Hierbei sollte auf qualitätsgesicherte und bedarfsorientierte, evidenzbasierte Angebote zurückgegriffen werden, die informativ und interaktiv gestaltet sind [27, 45].

## Praktische Umsetzungshinweise

Für eine gesundheitsförderliche Gestaltung von Arbeit im Homeoffice gibt es keine generalisierbare Lösung, die für alle Unternehmen und Situationen gleichermaßen passt.

Im Rahmen der Gefährdungsbeurteilung sollen psychische Belastungen überprüft werden

Daher sollten organisations- und tätigkeitsspezifische Lösungen erarbeitet und im Idealfall im Rahmen der Gefährdungsbeurteilung psychischer Belastungen nach § 5 ArbSchG überprüft werden. Die Gemeinsame Deutsche Arbeitsschutzstrategie (GDA) hat Empfehlungen für die Beurteilung der psychischen Belastungsfaktoren herausgegeben [18].

Bei der Umsetzung von Maßnahmen zur gesundheitsförderlichen Gestaltung der Homeoffice-Tätigkeit können Betriebe Beratung oder Unterstützung von folgenden Akteuren erhalten:

- Arbeitsmediziner/innen, Betriebsärzte/innen, Arbeitspsychologen/innen,
- psychologisch geschulte/ausgerichtet Fachkräften für Arbeitssicherheit,
- Erfahrungsaustausch innerhalb des Unternehmens,
- Unternehmensberatung, Beratungsfirmen für Betriebliche Gesundheitsförderung,
- für den Arbeitsschutz zuständige Behörden,
- zuständiger Unfallversicherungsträger,
- Krankenkassen, BGF-Koordinierungsstellen.

## Fazit für die Praxis

- Aufgrund der gesundheitlichen Gefahren der Verbreitung des neuartigen SARS-CoV-2-Virus und den damit einhergehenden Empfehlungen zur Kontaktreduktion greifen viele Unternehmen auf die Möglichkeit zurück, ihre Mitarbeiter/innen im Homeoffice zu beschäftigen.
- Für eine gesundheitsfördernde Gestaltung der Homeoffice-Tätigkeit im Hinblick auf das Vorbeugen sozialer Isolation sind die technischen Gegebenheiten (inklusive technischer Unterstützung) eine Grundvoraussetzung für einen beständigen Austausch.
- Eine beständige Kommunikation fördert soziale Unterstützung durch Kolleg/innen und Führungskräfte.
- Gesundheitsfördernde Führung von digital zusammenarbeitenden Teams zeichnet sich durch einen engen Kontakt zum Team, regen Informationsaustausch, Feedback und Vertrauen sowie einen partizipativen Führungsstil aus.
- Der Betrieb kann zudem zu individuell gesundem Verhalten unter sozial isolierten Umständen anregen und hierfür die entsprechenden Rahmenbedingungen schaffen.
